# Complicated intra-abdominal infections worldwide: the definitive data of the CIAOW Study

**DOI:** 10.1186/1749-7922-9-37

**Published:** 2014-05-14

**Authors:** Massimo Sartelli, Fausto Catena, Luca Ansaloni, Federico Coccolini, Davide Corbella, Ernest E Moore, Mark Malangoni, George Velmahos, Raul Coimbra, Kaoru Koike, Ari Leppaniemi, Walter Biffl, Zsolt Balogh, Cino Bendinelli, Sanjay Gupta, Yoram Kluger, Ferdinando Agresta, Salomone Di Saverio, Gregorio Tugnoli, Elio Jovine, Carlos A Ordonez, James F Whelan, Gustavo P Fraga, Carlos Augusto Gomes, Gerson Alves Pereira, Kuo-Ching Yuan, Miklosh Bala, Miroslav P Peev, Offir Ben-Ishay, Yunfeng Cui, Sanjay Marwah, Sanoop Zachariah, Imtiaz Wani, Muthukumaran Rangarajan, Boris Sakakushev, Victor Kong, Adamu Ahmed, Ashraf Abbas, Ricardo Alessandro Teixeira Gonsaga, Gianluca Guercioni, Nereo Vettoretto, Elia Poiasina, Rafael Díaz-Nieto, Damien Massalou, Matej Skrovina, Ihor Gerych, Goran Augustin, Jakub Kenig, Vladimir Khokha, Cristian Tranà, Kenneth Yuh Yen Kok, Alain Chichom Mefire, Jae Gil Lee, Suk-Kyung Hong, Helmut Alfredo Segovia Lohse, Wagih Ghnnam, Alfredo Verni, Varut Lohsiriwat, Boonying Siribumrungwong, Tamer El Zalabany, Alberto Tavares, Gianluca Baiocchi, Koray Das, Julien Jarry, Maurice Zida, Norio Sato, Kiyoshi Murata, Tomohisa Shoko, Takayuki Irahara, Ahmed O Hamedelneel, Noel Naidoo, Abdul Rashid Kayode Adesunkanmi, Yoshiro Kobe, Wataru Ishii, Kazuyuki Oka, Yoshimitsu Izawa, Hytham Hamid, Iqbal Khan, AK Attri, Rajeev Sharma, Juan Sanjuan, Marisol Badiel, Rita Barnabé

**Affiliations:** 1 Department of Surgery, Macerata Hospital, Macerata, Italy; 2 Emergency Surgery, Maggiore Parma Hospital, Parma, Italy; 3 Department of General Surgery, Ospedali Riuniti, Bergamo, Italy; 4 Department of Anestesiology, Ospedali Riuniti, Bergamo, Italy; 5 Department of Surgery, Denver Health Medical Center, Denver, USA; 6 American Board of Surgery, Philadelphia, USA; 7 Division of Trauma, Emergency Surgery and Surgical Critical Care, Harvard Medical School, Massachusetts General Hospital, Massachusetts, USA; 8 Department of Surgery, UC San Diego Health System, San Diego, USA; 9 Department of Primary Care & Emergency Medicine, Kyoto University Graduate School of Medicine, Kyoto, Japan; 10 Department of Abdominal Surgery, University Hospital Meilahti, Helsinki, Finland; 11 Department of Surgery, University of Newcastle, Newcastle, NSW, Australia; 12 Department of Surgery, Govt Medical College and Hospital, Chandigarh, India; 13 Department of General Surgery, Rambam Health Care Campus, Haifa, Israel; 14 Department of Surgery, Adria Hospital Adria, Adria, Italy; 15 Trauma Surgery Unit, Maggiore Hospital, Bologna, Italy; 16 Department of Surgery, Maggiore Hospital, Bologna, Italy; 17 Department of Surgery, Fundación Valle del Lilí, Cali, Colombia; 18 Division of Trauma/Critical Care Department of Surgery Virginia Commonwealth University, Richmond, VA, USA; 19 Division of Trauma Surgery, Campinas University, Campinas, Brazil; 20 Department of Surgery, Monte Sinai Hospital, Juiz de Fora, Brazil; 21 Department of Surgery, Emergency Unit, Ribeirão Preto, Brazil; 22 Department of Surgery, Chang Gung Memorial Hospital, Taoyuan, Taiwan; 23 Department of General Surgery, Hadassah Medical Center, Jerusalem, Israel; 24 Department of Surgery, Tianjin Nankai Hospital, Nankai Clinical School of Medicine, Tianjin Medical University, Tianjin, China; 25 Department of Surgery, Pt BDS Post-graduate Institute of Medical Sciences, Rohtak, India; 26 Department of Surgery, MOSC Medical College, Cochin, India; 27 Department of Surgery, SKIMS, Srinagar, India; 28 Department of Surgery, Kovai Medical Center, Coimbatore, India; 29 First Clinic of General Surgery, University Hospital/UMBAL/St George Plovdiv, Plovdiv, Bulgaria; 30 Department of Surgery, Edendale Surgery, Pietermaritzburg, Republic of South Africa; 31 Department of Surgery, Ahmadu Bello University Teaching Hospital Zaria, Kaduna, Nigeria; 32 Department of Surgery, Mansoura University Hospital, Mansoura, Egypt; 33 Department of Surgery, Faculdades Integradas Padre Albino, Catanduva, Brazil; 34 Department of Surgery, Mazzoni Hospital, Ascoli Piceno, Italy; 35 Department of Surgery, Mellini Hospital, Chiari, BS, Italy; 36 Department of General and Digestive Surgery, Virgen de la Victoria, University Hospital, Malaga, Spain; 37 Department of General Surgery and Surgical Oncology, Université de Nice Sophia-Antipolis, Universitary Hospital of Nice, Nice, France; 38 Department of Surgery, Hospital and Oncological Centre, Novy Jicin, Czech Republic; 39 Department of General Surgery, Lviv Emergency Hospital, Lviv, Ukraine; 40 Department of Surgery, University Hospital Center Zagreb, Zagreb, Croatia; 41 3rd Department of General Surger Jagiellonian Univeristy, Narutowicz Hospital, Krakow, Poland; 42 Department of Surgery, Mozyr City Hospital, Mozyr, Belarus; 43 Department of Surgery, Ancona University, Ancona, Italy; 44 Department of Surgery, Ripas Hospital, Bandar Seri Begawan, Brunei; 45 Clinical Sciences, Regional Hospitals Limbe and Buea, Limbe, Cameroon; 46 Department of Surgery, Severance Hospital, Yonsei University College of Medicine, Seoul, Republic of Korea; 47 Division of Trauma and Surgical Critical Care, Department of Surgery, University of Ulsan, Seoul, Republic of Korea; 48 II Cátedra de Clínica Quirúrgica, Hospital de Clínicas, Asuncion, Paraguay; 49 Department of Surgery, Cutral Có Clinic, Cutral Có, Argentina; 50 Department of Surgery, Faculty of Medicine Siriraj Hospital, Bangkok, Thailand; 51 Department of Surgery, Thammasat University Hospital, Pathumthani, Thailand; 52 Department of Surgery, Bahrain Defence Force Hospital, Manama, Bahrain; 53 Department of Surgery, Hospital Regional de Alta Especialidad del Bajio, Leon, Mexico; 54 Clinical and Experimental Sciences, Brescia Ospedali Civili, Brescia, Italy; 55 General Surgery, Adana Numune Training and Research Hospital, Adana, Turkey; 56 Visceral Surgery, Military Hospital Desgenettes, Lyon, France; 57 Visceral Surgery, Teaching Hospital Yalgado Ouedraogo, Ouedraogo, Burkina Faso; 58 Department of Acute and Critical care medicine, Tokyo Medical and Dental University, Tokyo, Japan; 59 The Shock Trauma and Emergency Medical Center, Matsudo City Hospital, Chiba, Japan; 60 Emergency and Critical Care Center of Nippon Medical School, Tama-Nagayama Hospital, Tokyo, Japan; 61 Department of Surgery, Our Lady of Lourdes Hospital, Drogheda, Ireland; 62 Department of Surgery, Port Shepstone Hospital, Port Shepstone, South Africa; 63 Department of Surgery, Obafemi Awolowo UNiversity Hospital, Ile-Ife, Nigeria; 64 Department of Emergency and Critical Care Medicine, Chiba University Hospital, Chiba, Japan; 65 Depatment of Emergency Medicine, Kyoto Second Red Cross Hospital, Kyoto, Japan; 66 Tajima emergency & Critical Care Medical Center, Toyooka Public Hospital, Toyooka, Hyogo, Japan; 67 Emergency and Critical Care Medicine, Jichi Medical University, Shimotsuke, Japan; 68 Department of Surgery, Mayo General Hospital Castlebar Co. Mayo, Castlebar, Ireland

## Abstract

The CIAOW study (Complicated intra-abdominal infections worldwide observational study) is a multicenter observational study underwent in 68 medical institutions worldwide during a six-month study period (October 2012-March 2013). The study included patients older than 18 years undergoing surgery or interventional drainage to address complicated intra-abdominal infections (IAIs).

1898 patients with a mean age of 51.6 years (range 18-99) were enrolled in the study. 777 patients (41%) were women and 1,121 (59%) were men. Among these patients, 1,645 (86.7%) were affected by community-acquired IAIs while the remaining 253 (13.3%) suffered from healthcare-associated infections. Intraperitoneal specimens were collected from 1,190 (62.7%) of the enrolled patients.

827 patients (43.6%) were affected by generalized peritonitis while 1071 (56.4%) suffered from localized peritonitis or abscesses.

The overall mortality rate was 10.5% (199/1898).

According to stepwise multivariate analysis (PR = 0.005 and PE = 0.001), several criteria were found to be independent variables predictive of mortality, including patient age (OR = 1.1; 95%CI = 1.0-1.1; p < 0.0001), the presence of small bowel perforation (OR = 2.8; 95%CI = 1.5-5.3; p < 0.0001), a delayed initial intervention (a delay exceeding 24 hours) (OR = 1.8; 95%CI = 1.5-3.7; p < 0.0001), ICU admission (OR = 5.9; 95%CI = 3.6-9.5; p < 0.0001) and patient immunosuppression (OR = 3.8; 95%CI = 2.1-6.7; p < 0.0001).

## Introduction

Intra-abdominal infections (IAIs) include a wide spectrum of pathological conditions, ranging from uncomplicated appendicitis to faecal peritonitis [1].

In the event of complicated IAI the infection proceeds beyond a singularly affected organ and causes either localized peritonitis (intra-abdominal abscesses) or diffuse peritonitis. Effectively treating patients with complicated intra-abdominal infections involves both source control and antimicrobial therapy [2,3].

In order to describe the epidemiological, clinical, microbiological, and surgical treatment profiles of complicated intra-abdominal infections (IAIs) in Europe, the World Society of Emergency Surgery (WSES) designed the CIAO Study (Complicated intra-abdominal infections observational study). The CIAO Study was conducted during 2012 across twenty European countries [4].

Given the interesting results of the CIAO Study, WSES designed a prospective observational study investigating the management of complicated intra-abdominal infections in a worldwide context.

The CIAOW study (Complicated intra-abdominal infections worldwide observational study) is a multicenter observational study underwent in 68 medical institutions worldwide during a six-month study period (October 2012-March 2013).

In January 2013 the preliminary results (2-month study period) of the CIAOW study were published [5].

WSES presents the definitive data of the CIAOW Study.

## Methods

### Aim

The purpose of the study was to describe the clinical, microbiological, and treatment profiles of both community- and healthcare-acquired complicated IAIs in a worldwide context.

Patients older than 18 years with both community-acquired and healthcare-associated IAIs were included in the database.

### Study population

The CIAOW study is a multicenter observational study underwent in 68 medical institutions worldwide. The study included patients undergoing surgery or interventional drainage to address complicated IAIs.

**Figure 1:**
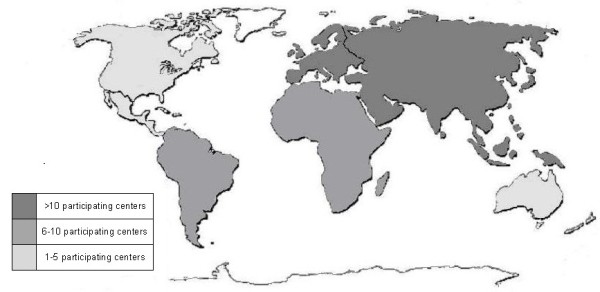
Participating centers for each continent.

Medical institutions from each continent participated in the study. The geographical distribution of the participating centers are represented in Figure 1.

### Study design

The study did not attempt to change or modify the laboratory or clinical practices of the participating physicians, and neither informed consent nor formal approval by an Ethics Committee were required.

The study met the standards outlined in the Declaration of Helsinki and Good Epidemiological Practices.

The study was monitored by the coordination center, which investigated and verified missing or unclear data submitted to the central database. This study was performed under the direct supervision of the board of directors of WSES.

### Data collection

In each centre, the coordinator collected and compiled data in an online case report system. These data included the following: (i) patient and disease characteristics, i.e., demographic data, type of infection (community- or healthcare-acquired), severity criteria, previous curative antibiotic therapy administered in the 7 days preceding surgery; (ii) origin of infection and surgical procedures performed; and (iii) microbiological data, i.e., identification of bacteria and microbial pathogens within the peritoneal fluid, the presence of yeasts (if applicable), and the antibiotic susceptibilities of bacterial isolates.

The primary endpoints included the following:

•Clinical profiles of intra-abdominal infections

•Epidemiological profiles (epidemiology of the microorganisms isolated from intra-abdominal samples and these organisms’ resistance to antibiotics)

•Management profiles

## Results

### Patients

2,020 cases were collected in the online case report system. 122 cases did not meet the inclusion criteria.

1,898 patients with a mean age of 51.6 years (range 18-99) were enrolled in the CIAOW study. 777 patients (41%) were women and 1,121 (59%) were men. Among these patients, 1,645 (86.7%) were affected by community-acquired IAIs while the remaining 253 (13.3%) suffered from heathcare-associated infections. Intraperitoneal specimens were collected from 1,190 (62.7%) of the enrolled patients [213 patients (84.2%) with Healthcare-associated infections and 977 (59.4%) with Community-acquired infections].

827 patients (43.6%) were affected by generalized peritonitis while 1071 (56.4%) suffered from localized peritonitis or abscesses.

296 patients (14.2%) were admitted in critical condition (severe sepsis/septic shock).

Table 1, 2 overview the clinical findings and radiological assessments recorded upon patient admission.

**Table 1:** Clinical findings

**Clinical findings**	**Patients**
	**N 1898 (100%)**
Abdominal pain	288 (15.1)
Abdominal pain, abdominal rigidity	284 (15%)
Abdominal pain, abdominal rigidity, T > 38°C or <36°C, WBC >12,000 or < 4,000	314 (16.5%)
Abdominal pain, abdominal rigidity, T > 38°C or <36°C,	67 (3.5)
Abdominal pain, abdominal rigidity, WBC >12,000 or < 4,000	376 (19.8%)
Abdominal pain, T > 38°C or <36°C,	68 (3.6%)
Abdominal pain, T > 38°C or <36°C, WBC >12,000 or < 4,000	139 (7.3%)
Abdominal pain, WBC >12,000 or < 4,000	266 (14%)
T > 38°C or <36°C	6 (0.3%)
T > 38°C or <36°C, WBC >12,000 or < 4,000	12 (0.6%)
Abdominal rigidity, WBC >12,000 or < 4,000	9 (0.5%)
Abdominal rigidity	2 (0.1%)
Abdominal rigidity, T > 38°C or <36°C	1 (0.05%)
Abdominal pain, abdominal rigidity, T > 38°C or <36°C, WBC >12,000 or < 4,000	7 (0.4%)
WBC >12,000 or < 4,000	11 (0.6%)
Not reported	48 (2.5%)

**Table 2:** Radiological procedures

**Radiological procedures**	**Patients**
	**N 1898 (100%)**
Abdomen X ray	240 (12.6%)
Abdomen X ray, CT	102 (5.4%)
Abdomen X ray, ultrasound	356 (18.7%)
Abdomen X ray, ultrasound, CT	112 (5.9%)
Abdomen X ray, ultrasound, MRI	4 (0.2%)
Abdomen X ray, CT,ultrasound, MRI	7 (0.4%)
CT	426 (22.4%)
CT, MRI	2 (0.1%)
Ultrasound	384 (20.2%)
Ultrasound, CT	87 (4.6%)
Ultrasound, CT, MRI	1 (0.05%)
Ultrasound, MRI	3 (0.1%)
MRI	1 (0.05%)
Not reported	173 (9.1%)

### Source control

The various sources of infection are outlined in Table 3. The most frequent source of infection was acute appendicitis; 633 cases (33.3%) involved complicated appendicitis.

**Table 3:** Source of infection

**Source of infection**	**Patients**
	**N 1898 (100%)**
Appendicitis	633 (33.3%)
Cholecystitis	278 (14.6%)
Post-operative	170 (15.,9%)
Colonic non diverticular perforation	115 (9.9%)
Gastroduodenal perforations	253 (13.3%)
Diverticulitis	106 (5.6%)
Small bowel perforation	145 (7.6%)
Others	122 (6.4%)
PID	30 (1.6%)
Post traumatic perforation	46 (2.4%)

The open appendectomy was the most common means of addressing complicated appendicitis. 358 patients (56.5%) admitted for complicated appendicitis underwent open appendectomies: 276 patients (77.1%) for localized infection or abscesses and 82 patients (22.9%) for generalized peritonitis. A laparoscopic appendectomy was performed for 226 patients (35.7%) with complicated acute appendicitis; of these patients, 193 (85.4%) underwent the procedure for localized peritonitis/abscesses and 33 (14.6%) underwent the procedure for generalized peritonitis.

Open bowel resection was performed for 5 patients affected by complicated appendicitis. In the other 48 cases of complicated appendicitis (7.6%), conservative treatment (percutaneous drainage, surgical drainage, and non-operative treatment) was performed. 3% of patients underwent percutaneous drainage (17/513) to address appendicular abscesses or localized intra-abdominal infections.

Among the patients with complicated cholecystitis (278), the open cholecystectomy was the most frequently performed procedure. 47.8% (133) and % 36.7 (102) of cholecystitis patients underwent open and laparoscopic cholecystectomies, respectively. The remaining patients were treated with conservative methods (percutaneous drainage, non-operative treatment).

Among the patients with complicated diverticulitis (106) the Hartmann resection was the most frequently performed procedure. 48 patients (45.3%) underwent a Hartmann resection. 31 of these patients (64.6%) underwent a Hartmann resection for generalized peritonitis, while the remaining 17 (35.6%) underwent the same procedure for localized peritonitis or abscesses. Colo-rectal resection was performed in 18 cases (17%) (5 with and 13 without protective stoma).

The remaining patients received conservative treatment (percutaneous drainage, non-operative treatment, surgical drainage and stoma). 4 patients underwent laparoscopic drainage.

For patients with gastro-duodenal perforations (253 cases), the most common surgical procedure was gastro-duodenal suture. 212 patients underwent open gastro-duodenal suture (83.8%) and 18 patients underwent laparoscopic gastro-duodenal suture (7.1%). 12 patients (4.7%) underwent gastro-duodenal resection and 6 patients (2.4%) received conservative treatment. The remaining patients underwent alternative procedures.

Of the 145 patients with small bowel perforations, 98 underwent open small bowel resection (85.2%) and 3 (2%) underwent laparoscopic small bowel resection. 28 patients (19.3%) were treated by stoma.

Among the 115 patients with colonic non-diverticular perforation, 42 (36.5%) underwent Hartmann resection, 26 (22.6%) underwent open resection with anastomosis and without stoma protection, and 26 underwent open resection with stoma protection (22.6%).

170 cases (8.9%) were attributable to post-operative infections.

Source control was successfully implemented for 1,735 patients (91.4%).

### Microbiology

Intraperitoneal specimens were collected from 1,190 patients (62.7%).

These specimens were obtained from 977 of the 1,645 patients presenting with community-acquired intra-abdominal infections (59.4%).

Intraperitoneal specimens were collected from 213 (84.2%) of the remaining 253 patients with nosocomial intra-abdominal infections.

The aerobic bacteria identified in intraoperative samples are reported In Table 4, 5.

**Table 4:** Aerobic bacteria identified from intra-operative peritoneal fluid

**Total**	**1.330 (100%)**
**Aerobic Gram-negative bacteria**	**957 (71.9%)**
Escherichia coli	548 (41.2%)
(Escherichia coli resistant to third generation cephalosporins)	75 (5.6%)
Klebsiella pneuumoniae	140 (10.5%)
(Klebsiella pneumoniae resistant to third generation cephalosporins)	26 (1.4%)
Klebsiella oxytoca	11 (0.8%)
(Klebsiella oxytoca resistant to third generation cephalosporins)	2 (0.1)
Enterobacter	64 (4.8%)
Proteus	47 (3.5%)
Pseudomonas	74 (5.6%)
Others	73 (5.6%)
**Aerobic Gram-positive bacteria**	**373 (29.1%)**
Enterococcus faecalis	153 (11.5%)
Enterococcus faecium	58 (4.4%)
Staphylococcus Aureus	38 (2.8%)
Streptococcus spp.	85 (6,4%)
Others	39 (2.9%)

**Table 5:** Aerobic bacteria from intra-operative samples in both community-acquired and healthcare-associated IAIs

**Community-acquired IAIs**	**Isolates n°**	**Healthcare-associated (nosocomial) IAIs**	**Isolates n°**
Aerobic bacteria	1030 (100%)	Aerobic bacteria	300 (100%)
Escherichia coli	456 (44.3%)	Escherichia coli	92 (21%)
(Escherichia coli resistant to third generation cephalosporins)	56 (5.4%)	(Escherichia coli resistant to third generation cephalosporins)	19 (6.3%)
Klebsiella pneumoniae	105 (10.1%)	Klebsiella pneumoniae	35 (11.7%)
(Klebsiella pneumoniae resistant to third generation cephalosporins)	11 (0.1%)	(Klebsiella pneumoniae resistant to third generation cephalosporins)	15 (5%)
Pseudomonas	56 (5.4%)	Pseudomonas	18 (5.7%)
Enterococcus faecalis	106 (10.3%)	Enterococcus faecalis	47 (15.7%)
Enterococcus faecium	38 (3.7%)	Enterococcus faecium	20 (6.7%)

The microorganisms isolated in subsequent samples from peritoneal fluid are reported in Table 6.

**Table 6:** Microorganisms identified from subsequent peritoneal samples

**Total**	**268 (100%)**
**Aerobic Gram-negative bacteria**	**195 (72.7%)**
Escherichia coli	105 (41.8%)
(Escherichia coli resistant to third generation cephalosporins)	35 (13.%)
Klebsiella pneuumoniae	41 (15.3%)
(Klebsiella pneumoniae resistant to third generation cephalosporins)	13 (4.8%)
Pseudomonas	20 (7.4%)
Others	29 (10.8%)
**Aerobic Gram-positive bacteria**	**41 (15.3%)**
Enterococcus faecalis	16 (6%)
Enterococcus faecium	10 (3.4%)
Staphylococcus Aureus	7 (4%)
Others	8 (3%)
Bacteroides	8 (3%)
Candida albicans	17 (6%)
Non candida albicans	6 (2.2%)
Other yeats	2 (0.7%)

All the microorganisms isolated in both intraoperative and subsequent samples from peritoneal fluid are reported in Table 7.

**Table 7:** Total of microorganisms identified from both intraoperative and subsequent peritoneal samples

**Total**	**1826 (100%)**
**Aerobic Gram-negative bacteria**	**1152 (63%)**
Escherichia coli	653 (35.7%)
(Escherichia coli resistant to third generation cephalosporins)	110 (6%)
Klebsiella pneuumoniae	181 (9.9%)
(Klebsiella pneumoniae resistant to third generation cephalosporins)	39 (2.1%)
Klebsiella oxytoca	11 (0.6%)
(Klebsiella oxytoca resistant to third generation cephalosporins)	2 (0.1)
Enterobacter	75 (4.1%)
Proteus	52 (2.8%)
Pseudomonas	94 (5.1%)
Others	102 (5.6%)
**Aerobic Gram-positive bacteria**	**414 (22.7%)**
Enterococcus faecalis	169 (9.2%)
Enterococcus faecium	68 (3.7%)
Staphylococcus Aureus	46 (2.5%)
Streptococcus spp.	85 (4.6%)
Others	47 (2.6%)
**Anaerobes**	**141 (7.7%)**
Bacteroides	108 (5.9%)
(Bacteroides resistant to Metronidazole)	3 (0.2%)
Clostridium	11 (0.6%)
Others	22 (1.2%)
**Candida spp.**	**117 (6.4%)**
Candida albicans	90 (4.9%)
(Candida albicans resistant to Fluconazole)	2 (0.1%)
Non-albicans Candida	27 (1.4%)
(non-albicans Candida resistant to Fluconazole)	3 (0.1%)
Other yeats	2 (0.1%)

The major pathogens involved in intra-abdominal infections were found to be *Enterobacteriaceae.*

Among the intra-operative isolates, Extended-Spectrum Beta-Lactamase (ESBL)-producing *Escherichia coli* isolates comprised 13.7% (75/548) of all *Escherichia coli* isolates, while ESBL-positive *Klebsiella pneumoniae* isolates represented 18.6% (26/140) of all *Klebsiella pneumoniae* isolates. ESBL-positive *Enterobacteriaceae* were more prevalent in patients with healthcare associated infections IAIs than they were in patients with community-acquired IAIs. ESBL-positive *Escherichia coli* isolates comprised 20.6% (19/92) of all identified *Escherichia coli* isolates, while ESBL-positive *Klebsiella pneumoniae* isolates made up 42.8% (15/35) of all identified *Klebsiella pneumoniae* isolates.

Among all the microorganisms isolated in both intraoperative and subsequent samples from peritoneal fluid, there were 110 isolates of *Escherichia coli ESBL,* 39 isolates of Klebsiella pneumoniae ESBL, 2 isolates of Klebsiella Oxytoca ESBL. There were 5 isolates of *Klebsiella pneumoniae* resistant to Carbapenems.

Among the microorganisms isolated in the intraoperative samples, there were 74 isolates of *Pseudomonas aeruginosa*, comprising 5.6% of all aerobic identified bacteria isolates.

Among all the microorganisms isolated in both intraoperative and subsequent samples from peritoneal fluid, there were 94 isolates of *Pseudomonas aeruginosa*, comprising 5.1% of all identified bacteria isolates.

The 2 *Pseudomonas aeruginosa* strains resistant to Carbapenems were also obtained from nosocomial infections.

Among all the aerobic gram-positive bacteria identified in the intraoperative samples, *Enterococci* (*E. faecalis and E. faecium*) were the most prevalent, representing 15.9% of all aerobic isolates, and were identified in 211 cases. Although *Enterococci* were also present in community-acquired infections, they were more prevalent in healthcare-associated infections (31.7%: 67/211).

Among all the microorganisms isolated in both intraoperative and subsequent samples from peritoneal fluid Enterococci were 237/1826 (12.9%).

11 glycopeptide-resistant *Enterococci* were identified; 5 were glycopeptide-resistant *Enterococcus faecalis* isolates and 6 were glycopeptide-resistant *Enterococcus faecium* isolates.

Tests for anaerobes were conducted for 486 patients.

Identified anaerobic bacteria from intra-operative specimens are reported in Table 8.

**Table 8:** Anaerobic bacteria identified from intra-operative peritoneal fluid

**Anaerobes**	**133**
Bacteroides	100 (75%)
(Bacteroides resistant to Metronidazole)	3 (1.5%)
Clostridium	11 (8.2%)
Others	22 (16.5%)

Among all the microorganisms isolated in both intraoperative and subsequent samples from peritoneal fluid, 141 anaerobes were observed. The most frequently identified anaerobic pathogen was *Bacteroides*. 108 *Bacteroides* isolates were observed during the course of the study.

In Table 9 are illustrated Candida spp. isolated in intra-operative specimens.

**Table 9:** Candida isolates identified from intra-operative peritoneal fluid

**Candida spp.**	**94**
Candida albicans	73 (78.7%)
(Candida albicans resistant to Fluconazole)	2 (2.1%)
Non-albicans Candida	21 (19.1%)
(non-albicans Candida resistant to Fluconazole)	3 (3.2%)

Among all the microorganisms isolated in both intraoperative and subsequent samples from peritoneal fluid, 117 *Candida* isolates were collectively identified (6%). 90 were *Candida albicans* and 27 were *non-albicans Candida*.

### Outcome

The overall mortality rate was 10.5% (199/1898).

565 patients (29.8%) were admitted to the intensive care unit (ICU) in the early recovery phase immediately following surgery.

223 patients (11.7%) ultimately required additional surgeries. 62 (11.3%) of these patients underwent open abdominal procedures.

In the immediate post-operative clinical period 269 patients were critically ill (132 with septic shock, 137 with severe sepsis).

According to univariate statistical analysis of the data (Table 10), septic shock (OR = 14.9; 95%CI = 9.3-26.7; p < 0.0001) and severe sepsis (OR = 4.2; 95%CI = 2.8-6.3; p < 0.0001) upon hospital admission were both predictive of patient mortality.

**Table 10:** Univariate analysis: risk factors for occurrence of death during hospitalization

**Risk factors**	**Odds ratio**	**95%CI**	**p**
*Clinical condition upon hospital admission*			
Severe sepsis	27.6	15.9-47.8	<0.0001
Septic shock	14.6	8.7-24.4	<0.0001
Healthcare associated infection	3.1	2.2-4.5	<0.0001
*Source of infection*			
Colonic non-diverticular perforation	21	9.9-44.6	<0.0001
Small bowel perforation	125.7	29.1-542	<0.0001
Complicated diverticulitis	11	4.9-25.2	<0.0001
Post-operative infections	19.1	9.3-39.3	<0.0001
Delayed initial intervention	2.6	1.8-3.5	<0.0001
*Immediate post-operative clinical course*			
Severe sepsis	33.8	19.5-58.4	<0.0001
Septic shock	59.2	34.4-102.1	<0.0001
ICU admission	18.6	12-28.7	<0.0001
*Comorbidities*			
Malignancy	3.6	2.5-15.1	p < 0.0001
Immunosoppression	1.0	3.2-7.5	p < 0.0001
Serious cardiovascular disease	4.5	3.2-6.3	p < 0.0001

The setting of acquisition was also a variable found to be predictive of patient mortality (healthcare-associated infections: OR = 3.1; 95%CI = 2.2-4.5; p < 0.0001).

Among the various sources of infection, colonic non-diverticular perforation (OR = 21; 95%CI = 9.9-44.6 p < 0.0001), complicated diverticulitis (OR = 11; 95%CI = 4.9-25.2; p < 0.0001), small bowel perforation (OR = 14.3; 95%CI = 6.7-30.3; p < 0.0001) and post-operative infections (OR = 19.1; 95%CI = 9.3-39.3; p < 0.0001) were significantly correlated with patient mortality.

Mortality rates did not vary to a statistically significant degree between patients who received adequate source control and those who did not. However, a delayed initial intervention (a delay exceeding 24 hours) was associated with an increased mortality rate (OR = 3.6; 95%CI = 1.9-3.7; p < 0.0001).

The nature of the immediate post-operative clinical period was a significant predictor of mortality (severe sepsis: OR = 10.5; 95%CI = 24.0-66.0; p < 0.0001, septic shock: OR = 39.8; 95%CI = 6.4-17.5; p < 0.0001). Patients requiring ICU admission (OR = 12.9; 95%CI = 8.8-19.0; p < 0.0001) were also associated with increased mortality rates.

Also comorbidities were associated to patient mortality (Malignancy: OR = 3.6; 95%CI = 2.5-15.1; p < 0.0001, immunosuppression: OR = 1.0; 95%CI = 3.2-7.5; p < 0.0001, and serious cardiovascular disease: OR = 4.5; 95%CI = 3.2-6.3, p < 0.0001).

According to stepwise multivariate analysis (PR = 0.005 and PE = 0.001) (Table 11), several criteria were found to be independent variables predictive of mortality, including patient age (OR = 1.1; 95%CI = 1.0-1.1; p < 0.0001), the presence of small bowel perforation: OR = 2.8; 95%CI = 1.5-5.3; p < 0.0001), a delayed initial intervention (a delay exceeding 24 hours) (OR = 1.8; 95%CI = 1.5-3.7; p < 0.0001), ICU admission (OR = 5.9; 95%CI = 3.6-9.5; p < 0.0001) and patient immunosuppression (OR = 3.8; 95%CI = 2.1-6.7; p < 0.0001).

**Table 11:** Multivariate analysis: risk factors for occurrence of death during hospitalization

**Risk factors**	**Odds ratio**	**95%CI**	**p**
Age	3.3	2.2-5	<0.0001
Small bowel perforation	27.6	15.9-47.8	<0.0001
Delayed initial intervention	14.6	8.7-24.4	<0.0001
ICU admission	2.3	1.5-3.7	<0.0001
Immunosuppression	3.8	2.1-6.7	<0.0001

Stepwise multivariate analysis, PR = 0.005 E PE = 0.001 (Hosmer-Lemeshow chi2(8) = 1.68, area under ROC curve = 0.9465).

## Discussion

The CIAOW Study confirmed that acute appendicitis is the most common intra-abdominal condition requiring emergency surgery worldwide. According to the WSES 2013 guidelines for management of intra-abdominal infections, both open and laparoscopic appendectomies are viable treatment options for complicated appendicitis [6]. CIAOW Study results indicate that the open approach was used in most patients and it was the most common approach in the patients with complicated appendicitis.

For patients with peri-appendiceal abscesses, the proper course of surgical treatment remains a point of contention in the medical community. Although guidelines for the management of intra-abdominal infections commonly assert that patients with peri-appendiceal abscesses should be treated with percutaneous image-guided drainage [5]. Percutaneous drainage with or without interval appendectomy to treat peri-appendiceal abscess results in fewer complications and shorter overall length of stay [6-8]. Data from CIAOW Study indicate that few patients underwent this procedure for a peri-appenceal abscess.

Laparoscopic cholecystectomy versus open cholecystectomy question for acute cholecystitis has been extensively investigated. Several studies showed that early laparoscopic cholecystectomy resulted in a significantly reduced length of stay, no major complications, and no significant difference in conversion rates when compared with initial antibiotic treatment and delayed laparoscopic cholecystectomy [9-12].

The open cholecystectomy was the most common means of treating complicated cholecystitis; 47.8% (133) of the patients with complicated cholecystitis underwent this procedure. By contrast, 36.7% (102) underwent a laparoscopic procedure.

The optimal surgical management of colonic diverticular disease complicated by peritonitis remains a controversial issue. Hartmann’s resection has been considered the procedure of choice in patients with generalized peritonitis and remains a safe technique for emergency colectomy in perforated diverticulitis, especially in elderly patients with multiple co-morbidities [13]. More recently, some reports have suggested that primary resection and anastomosis is the preferred approach to diverticulitis, even in the presence of diffuse peritonitis [14,15].

According to CIAOW Study data, the Hartmann resection was the most frequently performed procedure to address both complicated diverticulitis and non-diverticular colonic perforations worldwide.

The significance of microbiological analysis of infected peritoneal fluid in community-acquired intra-abdominal infections has been debated in recent years.

Although the absence of impact of bacteriological cultures has been documented especially in appendicitis [16], in this era of the broad spread of resistant microorganisms such as nosocomial and community extended-spectrum b-lactamase (ESBL) Enterobacteriaceae, carbapenemase producing gram negatives, b lactam- and vancomycin resistant enterococci (VRE), the threat of resistance is a source of major concern for clinicians. Therefore the results of the microbiological analyses have great importance for the therapeutic strategy of every patients.

According to CIAOW Study data, intraperitoneal specimens were collected from 62.7% of patients with complicated intra-abdominal infections.

Intraperitoneal specimens were collected in 59.4% patients presenting with community-acquired intra-abdominal infections.

Intraperitoneal specimens were collected from 84.2% of the patients with nosocomial intra-abdominal infections.

In many clinical laboratories, species identification and susceptibility testing of anaerobic isolates are not routinely performed. Tests for anaerobes were conducted for 486 patients.

The major pathogens involved in community-acquired intra-abdominal infections are Enterobacteriaceae, *Streptococcus* species, and certain anaerobes (particularly B. fragilis).

The main resistance threat in intra.-abdominal infections is posed by ESBL-producing Enterobacteriaceae, which are becoming increasingly common in community-acquired infections [17,18].

According to CIAOW Study data, ESBL producers were the most commonly identified drug-resistant microorganism involved in IAIs.

Recent years have seen an escalating trend of *Klebsiella pneumoniae* Carbapenemase (KPC) production, which continues to cause serious multidrug-resistant infections around the world. The recent emergence of Carbapenem-resistant Enterobacteriaceae is a major threat to hospitalized patients [19].

5 identified isolates of *Klebsiella pneumoniae* proved resistant to Carbapenems.

Pseudomonas aeruginosa is one of the major nosocomial pathogens worldwide. It is intrinsically resistant to many drugs and is able to become resistant to virtually any antimicrobial agent.

The rate of *Pseudomonas aeruginosa* was 5.6% of all microorganisms isolated in the intra-operative samples. According to CIAOW study there was no significant difference between community and healthcare associate infections.

The 2 *Pseudomonas aeruginosa* strains resistant to Carbapenems were also obtained from nosocomial infections.

Enterococci are significant pathogens in intra-abdominal infections. Among multidrug Gram positive bacteria, Enterococci remain a challenge. The evolution of antimicrobial resistance in these organisms poses enormous challenges for clinicians when faced with patients affected with Enterococcus infections. Enterococcus infections are difficult to treat because of both intrinsic and acquired resistance to many antibiotics.

Enterococci (E. faecalis and E. faecium) were the most common Gram positive aerobic isolates.

Although Enterococci were also identified in community-acquired infections, they were far more prevalent in nosocomial infections.

In the last years there has been a significant increase in the incidence of invasive infections due to Candida species.

Although the epidemiological role of Candida spp. in nosocomial peritonitis is not yet defined, the clinical role is significant, because Candida isolation is normally associated to a poor prognosis [20].

In the CIAOW Study 117 Candida isolates were collectively identified (6%). 90 were Candida albicans and 27 were non-albicans Candida.

It is well known that patients with severe sepsis or septic shock may be complicated by high mortality rates. According to the CIAOW Study the overall mortality rate was 10.5% (199/1898).

29.8% of patients were admitted to the ICU in the early recovery phase immediately following surgery. In the immediate post-operative clinical period 269 patients were critically ill (132 with septic shock, 137 with severe sepsis).

The surgical treatment strategies following an initial emergency laparotomy have been debated in the last years.

The decision whether and when to perform a relaparotomy in secondary peritonitis is largely subjective and based on professional experience. Factors indicative of progressive or persistent organ failure during early postoperative follow-up are the best indicators for ongoing infection and associated positive findings at relaparotomy [21-23].

Relaparotomy strategies may include either a relaparotomy, when the patient's condition demands it ("relaparotomy on-demand"), or a planned relaparotomy with temporarily abdomen closure or open abdomen [24-27].

In the CIAOW Study 223 post-operative patients (11.7%) ultimately required additional surgeries. 62 (11.3%) of these patients underwent open abdominal procedures.

According to univariate statistical analysis of the data, septic shock and severe sepsis upon hospital admission were both predictive of patient mortality.

The setting of acquisition was also a variable found to be predictive of patient mortality (healthcare-associated infections).

Among the various sources of infection, colonic non-diverticular perforation, complicated diverticulitis, small bowel perforation and post-operative infections were significantly correlated with patient mortality.

Mortality rates did not vary to a statistically significant degree between patients who received adequate source control and those who did not. However, a delayed initial intervention (a delay exceeding 24 hours) was associated with an increased mortality rate.

The nature of the immediate post-operative clinical period was a significant predictor of mortality. Patients requiring ICU admission were also associated with increased mortality rates.

Also comorbidities were associated to patient mortality.

According to stepwise multivariate analysis, several criteria were found to be independent variables predictive of mortality, including patient age, the presence of small bowel perforation, a delayed initial intervention (a delay exceeding 24 hours), ICU admission and patient immunosuppression.

## Conclusion

Complicated intra-abdominal infections remain an important source of patient morbidity and are frequently associated with poor clinical prognoses, particularly for patients in high-risk categories.

Given the sweeping geographical distribution of the participating medical centers, the CIAOW Study gives an accurate description of the epidemiological, clinical, microbiological, and treatment profiles of complicated intra-abdominal infections worldwide.

## Competing interests

The authors declare that they have no competing interests.

## Authors’ contributions

MS designed the study and wrote the manuscript. FCo and DC performed statistical analysis. All authors participated in the study.
